# Clinicopathological Implications of Proteinuria after Long-Term Isolated Hematuria due to Thin Basement Membrane Nephropathy and Focal Segmental Glomerulosclerosis

**DOI:** 10.1155/2019/1627392

**Published:** 2019-12-17

**Authors:** Ryo Togashi, Yoshikazu Nemoto, Kaito Waki, Michito Nagura, Shigeyuki Arai, Yoshifuru Tamura, Yasutoshi Oshima, Fukuo Kondo, Ryuji Ohashi, Shunya Uchida, Shigeru Shibata, Yoshihide Fujigaki

**Affiliations:** ^1^Department of Internal Medicine, Teikyo University School of Medicine, Itabashi-ku, Tokyo, Japan; ^2^Department of Pathology, Teikyo University Hospital, Itabashi-ku, Tokyo, Japan; ^3^Department of Diagnostic Pathology, Nippon Medical School Musashikosugi Hospital, Kasasaki-City, Kanagawa, Japan

## Abstract

A 45-year-old obese man presented with persistent hematuria for 21 years. At the age of 37, he developed hypertension and proteinuria which later increased up to 1.6 g/g creatinine. Kidney biopsy revealed thin basement membrane nephropathy (TBMN) and focal segmental glomerulosclerosis (FSGS), which explained his urinary abnormalities. Although a subgroup of TBMN can be complicated by FSGS, his FSGS was associated with obesity because of its histological features. Reduction of body weight and increasing a dose of angiotensin-receptor blocker could transiently reduce the amount of proteinuria. Clinicopathological implications of proteinuria after long-term hematuria by TBMN and FSGS were further discussed.

## 1. Introduction

The common causes of isolated microscopic hematuria among children and young adults are IgA nephropathy, thin basement membrane nephropathy (TBMN) and Alport syndrome (AS) [1, 2]. It is well recognized that both IgA nephropathy and AS could develop proteinuria, hypertension and renal dysfunction, resulting in end-stage kidney disease (ESKD). Although the renal outcome was believed to be generally good in TBMN, recent studies showed that a subgroup of TBMN is at increased risk of ESKD due to the late onset of focal segmental glomerulosclerosis (FSGS) [3]. Therefore, patients with persistent isolated microscopic hematuria, whatever the cause among children and young adults, need long-term follow-up to monitor proteinuria, renal function and blood pressure to provide patients with proper treatment at an appropriate timing.

We herein report a 45-year-old obese man who presented with proteinuria after long-term persistent hematuria. Kidney biopsy revealed TBMN and FSGS. We judged that his FSGS was caused by obesity because of its histological features. Differential diagnoses of clinical situations involving co-occurrence of TBMN and FSGS were also discussed.

## 2. Case Report

A 45-year-old man presented with occult blood in urine since 24 years old. He began to treat hypertension at the age of 37, when he had microscopic hematuria and proteinuria of 0.4 g/g creatinine. Since proteinuria was gradually increased to 1.16 g/g creatinine, he was admitted to our hospital to evaluate urinary abnormalities. He did not experience macroscopic hematuria. He was full-term birth, but birth weight information could not be obtained. He had no family history of renal diseases. On admission, height and body weight were 177 cm and 98.7 kg, respectively. Body mass index (BMI) was 31.5 kg/m^2^. He had over 80 kg (BMI >25.5 kg/m^2^) since 15 years old. Blood pressure was 140/78 mmHg. He was treated with the dosage of 25 mg/day of losartan potassium, 5 mg/day of amlodipine OD and 20 mg/day of febuxostat. Physical examination was not remarkable. Urinary examination showed proteinuria of 0.7 g/g creatinine, red blood cell of 10-19/high power filed and positive red cell casts. Blood chemistry showed serum creatinine of 0.69 mg/dL, cystatin C of 0.74 mg/L, albumin of 4.1 g/dL, uric acid of 6.3 mg/dL, LDL-cholesterol of 144 mg/dL and HbA1c of 5.5%. The estimated glomerular filtration rate calculated by the revised serum creatinine–based Japanese equation [4] was 97.6 mL/min/1.73 m^2^. Immunological examination indicated no abnormal results including C-reactive protein, IgG, IgA, complement and anti-nuclear antibody. The electrocardiogram and chest X-ray were normal. Abdominal ultrasound detected normal shape and size in the kidneys.

**Figure 1:**
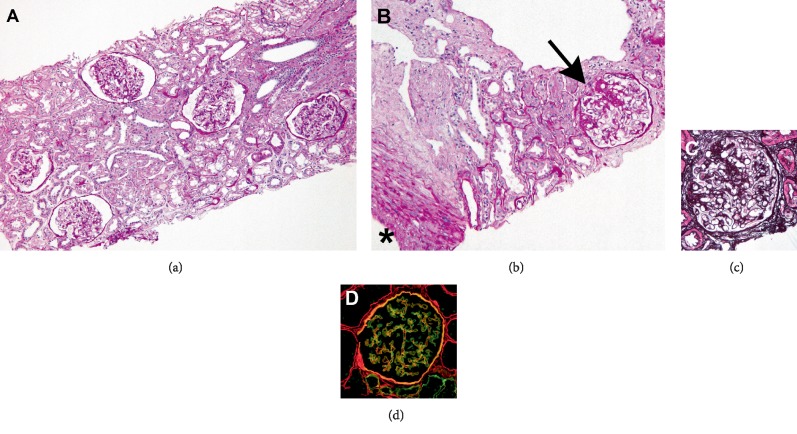
Kidney biopsy findings. (a): Light microscopic observation reveals glomeruli with slight enlargement. Periodic acid-Schiff staining. Original magnification ×200. (b) One glomerulus with focal segmental sclerosis is found at a corticomedullary junction area (arrow). ∗; arcuate artery. Periodic acid-Schiff staining. Original magnification ×200. (c) This glomerulus shows perihilar type of segmental sclerosis. Periodic acid-methenamine-silver staining. Original magnification ×200. (d) Immunofluorescent finding shows linear alpha-2 (red) and alpha-5 (green) of type IV collagen staining along the glomerular basement membrane. Yellow stain indicates merged image of co-localization of alpha-2 (red) and alpha-5 (green) of type IV collagen.

**Figure 2:**
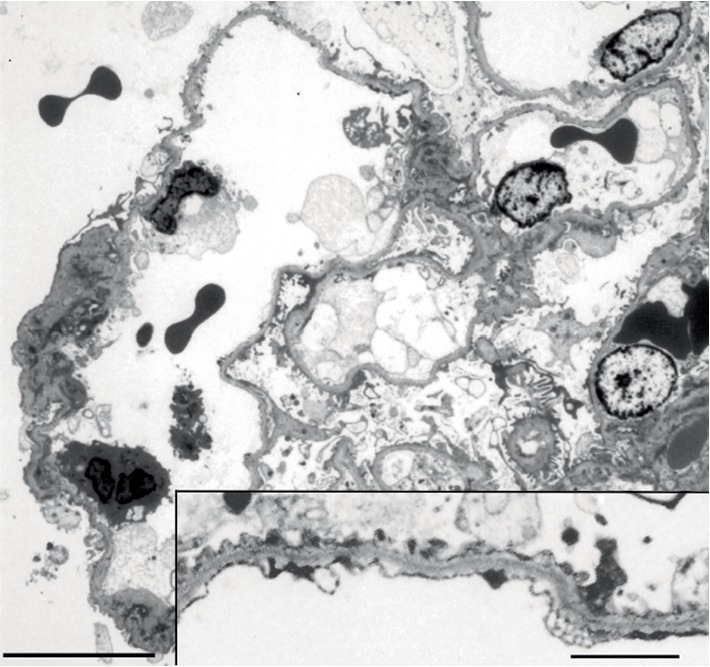
Electron micrograph of a glomerulus. Glomerular basement membranes are diffusely thinned without lamellation or reticular formation. Overlying podocytes are fairly preserved with focal foot process effacement. There are no electron dense deposits within glomerular capillary walls and a mesangial region. A red blood cell is seen in Bowman's space. Bar =10.0  *μ*m. Inset; Peripheral glomerular capillary wall. Bar =1.0 *μ*m.

With a clinical suspicion of IgA nephropathy, kidney biopsy was performed. Kidney biopsy revealed 1 global sclerosis out of 16 obtained glomeruli. Nonsclerotic glomeruli exhibited slight enlargement (glomerular diameter from 180 *μ*m to 200 *μ*m) without significant proliferative changes. One glomerulus showed segmental sclerosis at the perihilar region, diagnostic of FSGS (Figures 1(a)–1(c)). There were mild degree of tubular atrophy and interstitial fibrosis accompanied by mononuclear cell infiltration. Mild intimal thickening in small arteries and arteriolar hyalinosis were found. Immunofluorescent study showed no deposition of immnoreactants and a preserved linear alpha-5 of type IV collagen staining (Figure 1(d)), supportive for TBMN rather than AS [5]. Electron microscopy showed thinning of the glomerular basement membrane (GBM) (GBM width between 140 nm and 250 nm) without splitting or lamellation, fulfilling the diagnostic criteria of TBMN (Figure 2) [5]. Foot process effacement was partial, and glomerular epithelial detachment was not seen (Figure 2). Hypertension can be a cause of FSGS [6]. However, it is less likely that FSGS lesion in our patient was caused by hypertension because that his hypertension was less severe degree and that kidney biopsy did not include ischemic glomerular lesions, which are a common feature in hypertensive nephrosclerosis [7]. As our patient has significant obesity, the presence of glomerulomegaly and perihilar segmental glomerulosclerosis, and the absence of diffuse foot process effacement collectively supported the diagnosis of obesity-associated FSGS [8].

We further examined possible additional lesions consistent with obesity-related glomerulopathy (ORG). The glomerular density (the number of nonsclerotic glomeruli per renal cortical area) measured using an image analysis program (Aperio ImageScope, Leica, Buffalo Grove, IL, USA) was 1.7 mm^2^, which was low compared with that in nonobese kidney donors and comparable to the reported glomerular density in patients with ORG [9]. GBM width of the nonsclerotic area of the glomerulus with perihilar sclerosis was expected to be thick or normal in ORG [10], however, electron microscopic specimen including the glomerulus was not obtained. Since there was no detachment of podocytes from GBM, the expected reduction of the coating area of podocyte on the glomerular surface in ORG was not evident [8]. Other characteristics of ORG, such as tubular hypertrophy and the presence of the lipid droplets in the mesangial cells, podocytes and proximal tubular cells were not found [11].

Reduction of body weight by diet therapy from 98.7 kg to 92.0 kg, and increasing a dose of angiotensin-receptor blocker, losartan potassium to 100 mg/day successfully reduced blood pressure to 125/75 mmHg and proteinuria from 1.16 to 0.16 g/g creatinine 9 months after the kidney biopsy. However, subsequently re-increase in body weight brought about increasing proteinuria up to 1.2 g/g creatinine. Microscopic hematuria persisted, and renal function was stable during the clinical course.

## 3. Discussion

IgA nephropathy, TBMN and AS, all of three conditions can present with the manifestations of proteinuria after short or long-term isolated hematuria [1, 2]. Our patient with proteinuria after long-term hematuria had no family history of renal diseases and no visual or auditory deficits, thus IgA nephropathy was initially suspected. However, kidney biopsy revealed a dual diagnosis of TBMN and FSGS. The cause of FSGS needs to be clarified carefully in patients with such manifestations.

First, we postulated that FSGS might be a result of a co-inherited glomerulopathy with TBMN. TBMN is characterized by persistent isolated hematuria often in childhood or young adults. TBMN is usually autosomal dominant and caused by mutations in the genes encoding type IV collagen and heterozygous *COL4A3/COL4A4 *mutations may explain about 40% of familial microscopic hematuria due to TBMN [12]. Although the renal outcome was believed to be generally good, it is now recognized that up to 20–30% of patients of familial and sporadic TBMN cases with carriers of heterozygous *COL4A3/COL4A4 *mutations may progress to the ESKD due to late-onset FSGS often with nonnephrotic proteinuria [3, 12–14] or due to possible subset of AS [15]. Interestingly certain hypomorphic podocin variants may act as adverse genetic modifiers predisposing to FSGS when co-inherited with *COL4A3 or COL4A4 *mutations [16, 17]. No genotype-phenotype correlation was observed [18]. AS and TBMN have a common molecular basis. These conditions arise as a result of mutations in genes *COL4A3, COL4A4* and *COL4A5* that affect the synthesis, assembly, deposition or function of the collagen IV *α*345 molecules. Recently, the Alport Syndrome Classification Working Group proposed the classification of all disorders arising from abnormalities of the collagen IV *α*345 molecules as forms of AS with the goal of improving renal outcomes through regular monitoring and early treatment [19]. As our patient did not undergo genetic analysis, we were unable to determine if FSGS was caused by genetic abnormalities. Histopathologically, however, we believe that our FSGS was a secondary lesion because strong evidence of podocytopathy, such as diffuse process effacement and glomerular epithelial detachment, was absent by electron microscopic analysis.

Next, out of various factors leading to secondary FSGS, obesity was more likely to be the main cause because of our patient's morbid obesity. ORG is usually defined as glomerulomegaly with or without perihilar segmental glomerular sclerosis and partial foot process effacement in patients with BMI ≥30 kg/m^2^[8]. Typically ORG presents with either subnephrotic or less commonly nephrotic range proteinuria [8]. A glomerulomegaly with perihilar FSGS and relatively mild foot process effacement with moderate proteinuria can help to diagnose obesity-associated FSGS in our patient. However, it may not be easy to distinguish secondary FSGS from primary one from morphological aspects especially in the early phase of disease without any risk factors for secondary FSGS. As reported [8], reduction of body weight and reinforcement of renin angiotensin system inhibition were effective on reduction of proteinuria in our patient. This also supported a dual diagnose of TBMN and obesity-associated FSGS.

TBMN is not a rare disease since about 1% of the population is affected [20]. However, there is no evidence that TBMN alters the prognosis of another glomerulopathy [21]. On the other hand, obesity seems to worsen the renopathological state in chronic kidney disease [8, 22]. It is largely unknown whether obesity-associated FSGS or hypertension-associated FSGS is easy to develop in patients with TBMN. Given that the incidence of obesity and ORG has been increasing worldwide, we predict that more cases with a dual diagnose of TBMN and obesity-associated FSGS might be reported in the future [8, 23].

In summary, we experienced a patient with proteinuria after long-term isolated hematuria, diagnosed as TBMN complicated by secondary FSGS associated with obesity. Analysis of *COL4A3* or *COL4A4 *mutations might be helpful to know the underlying risk not only for FSGS but also for AS in patients with long-term isolated hematuria [3, 15]. However, genetic diagnosis that can predict a progression of FSGS in patients with TBMN has not been established, therefore careful evaluation of clinicopathological findings is essential for a proper diagnosis and an appropriate treatment.

## Additional Points

This article does not contain any studies with human participants performed by any of the authors.
